# Examining the relationship between nurse psychological capital and job burnout: a multilevel analysis across nurse, nurse leader, and nurse family perspectives

**DOI:** 10.1186/s12960-025-00986-5

**Published:** 2025-03-28

**Authors:** Mengjie Xia, Junqiang Wang, Zhibin Wang, Dongjun Bi, Huiping Mao, Xiaohong Liu, Lili Feng, Chen Lili, Xiaoting Yan, Fang Huang, Rusli Nordin, Zainooriah Dato’ Hj. Zakaria

**Affiliations:** 1 School of Medicine, Taizhou University, Jiaojiang, Zhejiang, 318000 China; 2 School of Nursing, Faculty of Medicine Bioscience & Nursing, MAHSA University, 42610 Jenjarom, Selangor Malaysia; 3 Department of Obstetrics and Gynecology, Taizhou Hospital of Zhejiang Province Affiliated to Wenzhou Medical University, Luqiao, Zhejiang, 318050 China; 4 School of Teacher Education (Physical Education), Taizhou University, Linhai, Zhejiang, 317000 China; 5 Department of Nursing, Taizhou Hospital of Zhejiang Province Affiliated to Wenzhou Medical University, Luqiao, Zhejiang, 318050 China; 6 Department of Nursing, Taizhou First People’s Hospital, Huangyan, Zhejiang, 318020 China; 7 Department of Nursing, Taizhou Central Hospital (Taizhou University Hospital), Jiaojiang, Zhejiang, 318000 China

**Keywords:** Job burnout, Psychological capital, Nurse, Nurse leader, Nurse family

## Abstract

**Background:**

Nurse job burnout is a critical issue affecting medical quality and safety. Psychological capital (PsyCap) is associated with enhanced career satisfaction and reduced work stress. This study evaluates the relationship between nurses’ PsyCap and job burnout, while considering leadership and family as contextual factors.

**Methods:**

A cross-sectional study design was used, collecting data from 499 nurses via validated questionnaires. Statistical analyses, including descriptive statistics, correlation, and multiple regression, were conducted to assess the relationship between PsyCap and job burnout. Leadership and family were included as contextual variables in hierarchical regression models to evaluate their indirect influences.

**Results:**

Among nurses, 63.9% experienced mild to moderate burnout. The average PsyCap score was 107.88 ± 20.55. PsyCap showed a significant negative correlation with burnout dimensions (correlation coefficients: −0.43 to −0.53, p < 0.01). Higher PsyCap significantly predicted lower job burnout (β = −0.44, p < 0.01). Leadership and family influences had minor but noteworthy indirect effects on this relationship.

**Conclusion:**

The results of this study suggest that higher levels of nurses’ PsyCap are associated with lower levels of job burnout. While these findings highlight PsyCap’s potential role in mitigating burnout, further research is needed to confirm causal relationships and assess the effectiveness of interventions aimed at enhancing PsyCap and supportive environments.

## Background

Nurses are core members of the healthcare team, directly involved in patient care and influencing patient recovery outcomes and satisfaction [1]. However, nurses often experience job burnout due to constant work pressure, high labor intensity, and complex interpersonal relationships [2]. It can also reduce work efficiency and quality of care, affecting the operational efficiency of the entire healthcare organization and patient safety [3, 4].

In recent years, psychological capital (PsyCap) has emerged as a key construct in management and psychological research [5, 6]. PsyCap, which includes four dimensions of self-efficacy, hope, resilience, and optimism, has been shown to increase job satisfaction and performance while reducing job burnout [7, 8]. It represents an internal psychological resource that empowers nurses to navigate work-related challenges, maintain positivity, and excel in their roles [9].

Although prior research has primarily focused on the individual relationship between PsyCap and burnout, broader contextual influences remain underexplored [6, 8]. Leadership and family support are particularly relevant in the nursing profession, where interpersonal and systemic factors shape workplace dynamics [8]. Nurse leaders play a crucial role in creating supportive work environments, while family support serves as a critical foundation for nurses’ personal resilience [10, 11].

This study investigates the relationship between nurses’ PsyCap and job burnout, addressing existing gaps in the literature by incorporating leadership and family support as contextual influences. Conducted in diverse healthcare settings across Zhejiang Province, China, the study aims to provide a nuanced understanding of PsyCap’s role in mitigating burnout and offers practical insights for developing targeted interventions.

## Methods

### Study design

This study employed a cross-sectional design to investigate the relationship between nurse PsyCap and job burnout. Nurse leaders’ and family members’ PsyCap were treated as contextual factors and incorporated as control variables in the analysis to account for their indirect influence on nurses’ PsyCap and job burnout.

### Setting and participants

This study was conducted in three provincial and municipal hospitals and three township health centers in Taizhou City, Zhejiang Province, China. These facilities were selected based on their representativeness of healthcare settings in urban and rural China. Provincial and municipal hospitals were chosen for their advanced infrastructure, high patient volumes, and diverse nursing teams, representing high-pressure work environments. Township health centers were included to capture the experiences of nurses working in resource-limited, community-focused settings, offering a comprehensive comparison of urban and rural nursing contexts.

### Recruitment process

Recruitment for this study utilized a multi-faceted approach to ensure representation across nurses, nurse leaders, and family members. Nurses were recruited via their hospital nursing departments. Department administrators distributed an email invitation containing study details and a link to the online questionnaire. Participation was voluntary, and informed consent was obtained.

Nurse leaders who directly supervised the recruited nurses were contacted through their departments. An invitation email and survey link were shared to align their participation with that of the nurses they oversaw.

Family members were invited by participating nurses to ensure a variety of familial relationships (spouses, parents, siblings, or adult children). A clear explanation of the study purpose and confidentiality assurances were provided to encourage independent participation and minimize self-selection bias.

### Inclusion and exclusion criteria

Nurses participating in this study were required to meet the following inclusion criteria: (1) hold a valid certificate of nursing practice issued by the People’s Republic of China, (2) have at least one year of professional nursing experience, and (3) be actively employed at the time of data collection. Exclusion criteria included the following: (1) current participation in further education programs, (2) extended sick leave or maternity leave, (3) pregnancy at the time of the study, and (4) Self-reported diagnoses of severe mental health conditions, such as clinical depression or anxiety disorders, within the past 12 months. These criteria aimed to minimize confounding factors that could influence PsyCap and job burnout. Severe mental health conditions were defined as psychiatric diagnoses requiring medical intervention, which might affect baseline PsyCap levels or amplify burnout, complicating the analysis.

Nurse leaders were included if they (1) held a leadership position for at least three years, (2) were actively employed during the study period, and (3) had direct supervisory responsibilities over nurses participating in the study. Similar to nurses, nurse leaders were excluded if they were on extended leave or self-reported severe mental health conditions in the past year.

Family members of nurses were included in this study if they (1) had a close familial relationship with the participating nurse, such as a spouse, parent, sibling, or adult child (aged 18 years or older), and (2) were willing to participate in the study after being informed of its purpose. Family members were excluded if they (1) were not in regular contact with the participating nurse or (2) self-reported severe mental health conditions, such as clinical depression or anxiety disorders, within the past 12 months. These criteria were designed to ensure that family members included in the study could provide meaningful insights into their PsyCap and its potential influence on the participating nurses.

### Nurse sample size estimation

The nurse sample size was calculated using the Scalex SP calculator [12]. Considering an expected prevalence of 65% [13] and a desired margin of error of ± 5% with 95% confidence, a sample size of 438 was determined. This sample size accounted for a potential loss of 20% and provided an anticipated 95% confidence interval of (60%, 70%).

### Sampling method and final sample characteristics

The study sampled the nurse population from three provincial and municipal hospitals in Zhejiang Province using a stratified random sampling strategy. This method ensured that the samples were representative of each hospital’s nurse population according to its size [14]. The total population of nurses in the three hospitals was 2,396, with tertiary hospitals sampling 35%, 34%, and 31% of the population respectively based on headcount. Sample selection was conducted using sampling software, which randomly selected nurses in each hospital based on their work number to ensure a fair and impartial sampling process. For township health centers, given their small size, all nurses were included using the whole number survey method to get a comprehensive picture of PsyCap and job burnout among nurses.

Nursing leaders who were in direct contact with nurses were selected for the survey using a quota sampling technique to ensure the representation of all relevant subgroups. The sample of nurses’ family members was sampled using a convenience sampling methodology that included a variety of family relationships to provide a comprehensive perspective on the impact of family on nurses’ psychological well-being. The link to the questionnaire for nurse leaders was shared by the nursing department to the nurse leader of the participating nurse’s unit, and the link for the nurse’s family members was shared by the nurse with the questionnaire link.

After eliminating 15 invalid questionnaires, the final sample consisted of 278 nurses from provincial and municipal hospitals and 221 nurses from township health centers. The nursing leader group consisted of 108 nursing leaders directly related to the work of nurses, including 53 from provincial and municipal hospitals and 55 from township health centers. The sample of nurses’ family members was diverse, including 237 spouses, 95 siblings, 80 parents and 20 children (over 18 years of age).

### Research instruments

#### Measurement of PsyCap

To assess the PsyCap of nurses, nurse leaders, and family members, the Psychological Capital Questionnaire (PCQ-24) translated by Li Chaoping and compiled by Luthans [15, 16] was utilized. This instrument comprises 24 items, categorized under four dimensions, and each dimensions has 6 items: self-efficacy, hope, resilience, and optimism. Participants were instructed to rate each statement on a scale ranging from 1 (strongly disagree) to 6 (strongly agree). A score of 5 or more usually indicates strong PsyCap. Higher scores on this scale indicate greater PsyCap, thus providing an overall picture of the psychological assets and competencies of nurses, nurse leaders, and their families. The PCQ-24 has been validated for its applicability to the Chinese nursing community [17–19]. In this investigation, Cronbach’s α coefficients are 0.975 for nurses and nurse leaders and 0.961 for family members. The subsequent confirmatory factor analysis (CFA) for nurses indicated: χ2/df = 6.07, GFI = 0.89, CFI = 0.90, RMSEA = 0.10, and RMR = 0.05. For nurse leaders, the CFA metrics were: χ2/df = 2.11, GFI = 0.83, CFI = 0.90, RMSEA = 0.10, and RMR = 0.05. Meanwhile, the CFA results for the family members of nurses were: χ2/df = 5.05, GFI = 0.87, CFI = 0.90, RMSEA = 0.10, and RMR = 0.10.

#### Measurement of job burnout

The Chinese version of the Maslach Burnout Inventory-General Survey (MBI-GS), developed by Maslach et al. and revised by Li Chaoping [20, 21], was employed to assess job burnout. The scale consists of 15 items, categorized into three dimensions: emotional exhaustion (5 items), cynicism (4 items), and reduced professional efficacy (6 items). Responses ranged from 0 (never) to 6 (every day). The overall job burnout index was computed as: total scores = emotional exhaustion*0.4 + cynicism*0.3 + reduced professional efficacy*0.3 [22]. Interpretation of the cumulative score was as follows: 0—1.49 indicated no job burnout; 1.50—3.49 suggested mild to moderate job burnout, and scores from 3.50—6 were indicative of severe job burnout. The Chinese-adapted MBI-GS boasts solid validity and commendable internal consistency [23, 24]. In this research, its reliability (Cronbach’s α) was measured at 0.966, and the CFA reported: χ2/df = 6.92, GFI = 0.94, CFI = 0.95, RMSEA = 0.11, and RMR = 0.09.

### Quality control

To ensure data integrity, this study implemented several protocols. During preparation, the research team managed the study’s design and provided standardized training to investigators on survey objectives and administration. During the implementation phase, participants received a brief introduction, informed consent was obtained, and their responses were carefully monitored to ensure completeness. For data transcription, a double-entry system was adopted with independent checks for consistency and accuracy.

### Data collection

In this study, nurses, nurse leaders, and nurse family members served as participants. Data was collected via the Questionnaire Star software. While nurse leaders and family members responded to queries about PsyCap, nurses addressed questions on both PsyCap and job burnout. Each nurse fills in the hospital and working number as a unique identifier to facilitate the distribution of the questionnaire to leaders and family members, while matching with the corresponding nurse entry on the platform. To enhance family member participation, the questionnaire clarified that respondents could be close relatives, such as spouses, parents, siblings, or children.

### Data analysis

Data analysis was performed using SPSS 26.0 (IBM Corp., Armonk, NY, USA). Initially, descriptive statistics were computed to provide means and standard deviations for all primary variables. A one-way analysis of variance (ANOVA) was employed to investigate the potential differences in scale outcomes based on demographic variables. Spearman correlations were used to examine the relationships between the PsyCap of the three groups (nurses, nurse leaders, and nurses’ family members) and nurses’ job burnout. In the multiple linear regression model, the PsyCap scores of nurses, nurse leaders, and family members were included to examine their independent and combined effects on nurse job burnout. The inclusion of these three variables follows a multi-level approach to understanding burnout, acknowledging that while individual psychological resources (nurse PsyCap) are critical, both leadership (nurse leader PsyCap) and family support (family PsyCap) provide important contextual support. Lastly, to delve deeper into the mediating role of nurses’ PsyCap between the PsyCap of nursing leaders and family members and nurses’ job burnout, a mediation effect analysis was carried out using the PROCESS macro.

## Result

### Descriptive statistics

In this study cohort of 499 nurses, 63.9% (319 nurses) reported experiencing some level of job burnout. Among them, 44.9% (224 nurses) reported mild to moderate burnout, while 19% (95 nurses) reported severe burnout. The average MBI-GS score across all nurses was 2.22 (SD = 1.39), indicating a mild to moderate level of burnout. The average PsyCap score was 107.88 (SD = 20.55).

Table 1 shows MBI-GS and PCQ scores across demographic and professional attributes. Nurses in provincial and municipal hospitals reported lower MBI-GS scores (M = 2.12, SD = 1.34) and higher PCQ scores (M = 111.19, SD = 19.17) compared to nurses in township health centers (MBI-GS: M = 2.35, SD = 1.36; PCQ: M = 103.72, SD = 21.48), with significant differences in PCQ scores (p < 0.01). Gender-based analysis showed that female nurses, comprising 96.8% of the sample, reported higher burnout levels (MBI-GS: M = 2.24, SD = 1.39) than male nurses (MBI-GS: M = 1.44, SD = 0.94), while male nurses exhibited stronger PsyCap scores (M = 115.94, SD = 15.14, p < 0.05).

**Table 1:** Comparative analysis of MBI-GS and PCQ scores across demographic and professional characteristics of nurses

Category	Subcategory	N (%)	Calculated MBI-GS mean (SD)	PCQ Total Score mean (SD)	Welch's Test P-value (MBI-GS/PCQ)
Hospital Types					0.066*/0.000***
	Provincial and municipal hospital	278 (55.71)	2.12 (1.34)	111.19 ( 19.17)	
	Township health center	221 (44.29)	2.35 (1.44)	103.72 ( 21.48)	
Gender					0.004***/0.048**
	Female	483 (96.79)	2.24 (1.39)	107.61 (20.66)	
	Male	16 (3.21)	1.44 (0.94)	115.94 ( 15.14)	
Age					0.006***/0.019**
	≥ 40	115 (23.05)	2.18 (1.55)	111.57 ( 21.24)	
	35–39	97 (19.44)	1.80 (1.33)	110.80 (20.88)	
	30–34	69 (13.83)	2.73 (1.25)	108.80 (18.75)	
	24–29	172 (34.47)	2.33 (1.36)	104.62 (19.54)	
	18–23	46 (9.22)	2.56 (1.22)	103.33 (22.35)	
Years of Work Experience					0.184/0.022**
	12 years and above	191 (38.28)	2.05 (1.46)	111.10 (21.06)	
	10–12 years	54 (10.82)	2.45 (1.49)	110.44 (20.13)	
	7–9 years	62 (12.43)	2.17 (1.28)	105.00 (21.95)	
	4–6 years	110 (22.04)	2.31 (1.38)	106.16 (18.71)	
	1–3 years	82 (16.43)	2.44 (1.19)	103.17 (19.84)	
Professional Title					0.532/0.006***
	Chief nurse	1 (0.20)	1.25 (0.00)	113.00 (0.00)	
	Associate chief nurse	14 (2.81)	2.05 (1.72)	120.64 (16.11)	
	Charge nurse	180 (36.07)	2.08 (1.43)	110.41 (21.93)	
	Nursing practitioner	179 (35.87)	2.29 (1.35)	106.79 (18.96)	
	Nurse	125 (25.05)	2.34 (1.35)	104.33 (20.38)	
Total		499 (100)	2.22 (1.39)	107.88 (20.55)	

*** *p* < .01; ** *p* < .05; * *p* < .10

*MBI-GS* = Maslach Burnout Inventory—General Survey; *PCQ* = Psychological Capital Questionnaire

Significant age-related trends were observed. Nurses aged 30–34 years exhibited the highest burnout levels (MBI-GS: M = 2.73, SD = 1.25), while those aged 35–39 reported the lowest (MBI-GS: M = 1.08, SD = 1.33; p < 0.01). Among professional titles, associate chief nurses had the highest PsyCap scores (M = 120.64, SD = 16.11; p < 0.01).

Nurse leaders’ demographic characteristics and PCQ scores are contrasted in Table 2. The emergency department leaders had the highest score at 128.50 (SD = 8.42), and outpatient clinic nurse leaders had the lowest level (M = 110.12, SD = 18.77, p < 0.05).

**Table 2:** Comparative analysis of PCQ scores across demographic and professional characteristics of nurse leaders

Category	Subcategory	N (%)	PCQ mean (SD)	Welch’s Test P-value (PCQ)
Hospital types				0.125
	Provincial and Municipal Hospital	53 (49.07)	118.66 (14.17)	
	Township Health Center	55 (50.93)	113.06 (22.65)	
Current department				0.009***
	Internal Medicine	26 (24.07)	115.85 (22.28)	
	Surgery	18 (16.67)	120.60 (18.86)	
	Emergency Department	8 (7.41)	128.50 (8.42)	
	Outpatient Clinic	17 (15.74)	110.12 (18.77)	
	ICU	10 (9.26)	120.60 (18.86)	
	Operating Room	9 (8.33)	125.67 (8.68)	
	Obstetrics and Gynecology	13 (12.04)	111.46 (18.50)	
	Pediatrics	7 (6.48)	111.00 (13.61)	
Total		108 (100.00)	115.81 (19.09)	

*** *p* < .01; ** *p* < .05; * *p* < .10

*PCQ* = Psychological Capital Questionnaire

Table 3 shows the demographic characteristics and PCQ scores of the nurses’ family members. Regarding the relationship with the nurse, significant variations were noted (p < 0.05), with parents showing the lowest PCQ score at 105.98 (SD = 15.92). Family members of nurses older than 40 years had the lowest PCQ scores (M = 106.19, SD = 15.67), and PCQ scores for other age groups were relatively closer (mean 114.52–116.67, p < 0.01) Education level also revealed significant disparities (p < 0.05), with master’s degree holders highest score of 114.32 (SD = 17.80). Years of work experience they highlighted significant variations (p < 0.01), with a peak score of 121.96 (SD = 17.02) for those with 4–6 years of experience.

**Table 3:** Comparative analysis of PCQ scores across demographic and professional characteristics of nurse families

Category	Subcategory	N (%)	PCQ mean (SD)	Welch's Test P-value (PCQ)
Relationship with the Nurse				0.020**
	Spouse	237 (54.86)	112.33 (17.83)	
	Siblings	95 (21.99)	113.23 (22.15)	
	Parents	80 (18.52)	105.98 (15.92)	
	Children	20 (4.63)	112.85 (15.80)	
Age				0.000***
	≥ 40	195 (45.14)	106.19 (15.67)	
	35–39	63 (14.58)	114.52 (18.23)	
	30–34	81 (18.75)	116.67 (17.08)	
	24–29	60 (13.99)	115.00 (24.67)	
	18–23	33 (7.64)	116.49 (19.00)	
Education Level				0.012**
	Master's Degree or above	22 (5.09)	114.32 (17.80)	
	Bachelor's Degree	202 (46.76)	111.37 (18.99)	
	Associate Degree	98 (22.69)	111.37 (18.99)	
	High School	38 (8.80)	106.78 (18.21)	
	Junior High School or below	72 (16.67)	106.94 (15.14)	
Years of Work Experience				0.000***
	12 years and above	218 (50.46)	108.73 (16.43)	
	10–12 years	51 (11.81)	107.66 (16.76)	
	7–9 years	49 (11.34)	119.59 (20.00)	
	4–6 years	48 (11.11)	121.96 (17.02)	
	1–3 years	20 (4.63)	96.65 (26.83)	
	Not yet employed	46 (10.65)	114.65 (17.66)	
Total		432 (100.00)	111.38 (18.58)	

*** *p* < .01; ** *p* < .05; * *p* < .10

PCQ = Psychological capital questionnaire

### Correlation analysis

The relationships between nurses’ PsyCap and job burnout dimensions are summarized in Table 4. Significant positive correlations were observed between the burnout dimensions of emotional exhaustion, cynicism, and reduced professional efficacy (r = 0.62 to 0.82, all p < 0.01). The total MBI-GS score strongly correlated with its subdimensions (r = 0.88 to 0.94, all p < 0.01), indicating that these components collectively influence overall burnout.

**Table 4:** Spearman correlations between nurse psychological capital and job burnout

Variables	1	2	3	4	5	6	7	8	9
1. N-EE	1***								
2. N-CY	0.82***	1***							
3. N-PE	0.62***	0.77***	1***						
4. N-MBI-GS	0.88***	0.94***	0.90***	1***					
5. N-Self-efficacy	−0.44***	−0.50***	−0.39***	−0.48***	1***				
6. N-Hope	−0.43***	−0.51***	−0.40***	−0.49***	0.83***	1***			
7. N-Resilience	−0.38***	−0.46***	−0.39***	−0.45***	0.76***	0.83***	1***		
8. N-Optimism	−0.43***	−0.50***	−0.41***	−0.48***	0.74***	0.79***	0.84***	1***	
9. N-PCQ	−0.45***	−0.53***	−0.43***	−0.51***	0.91***	0.93***	0.93***	0.90***	1***

*** *p* < .01; ** *p* < .05; * *p* < .10

*N*- = Nurse, *EE* = Emotional Exhaustion, *CY* = Cynicism, *PE* = Professional Efficacy

PsyCap components (self-efficacy, hope, resilience, and optimism) showed significant negative correlations with all burnout dimensions (r = −0.38 to −0.53, all p < 0.01), highlighting the protective role of PsyCap against burnout. Additionally, strong positive interrelations were observed among PsyCap components (r = 0.74 to 0.84, all p < 0.01), reflecting their collective contribution to psychological resilience.

For nurse leaders (Table 5), the correlation between their PsyCap and nurses’ burnout dimensions was weak and non-significant (p > 0.05), suggesting limited direct influence. In contrast, Table 6 shows that family members’ PsyCap exhibited weak but significant negative correlations with nurses’ emotional exhaustion (r =−0.19), cynicism (r = −0.18), and overall burnout scores (r = −0.14, p < 0.05).

**Table 5:** Spearman correlations between nurse leader psychological capital and nurse job burnout

Variables	1	2	3	4	5	6	7	8	9
1. N-EE	1***								
2. N-CY	0.82***	1***							
3. N-PE	0.62***	0.77***	1***						
4. N-MBI-GS	0.88***	0.94***	0.90***	1***					
5. NL-Self-efficacy	0.02	0.03	0.00	0.02	1***				
6. NL-Hope	−0.02	−0.01	−0.04	−0.03	0.70***	1***			
7. NL-Resilience	0.02	0.02	0.000	0.01	0.57***	0.72***	1***		
8. NL-Optimism	0.08*	0.07	0.07	0.08*	0.51***	0.57***	0.69***	1***	
9. NL-PCQ	0.05	0.05	0.03	0.05	0.68***	0.78***	0.86***	0.81***	1***

*** *p* < .01; ** *p* < .05; * p < .10

*N*- = Nurse; *NL*- = Nurse Leader; *EE* = Emotional Exhaustion; *CY* = Cynicism; *PE* = Professional Efficacy

**Table 6:** Spearman Correlations between Nurse Family Psychological Capital and Nurse Job Burnout

Variables	1	2	3	4	5	6	7	8	9
1. N-EE	1***								
2. N-CY	0.82***	1***							
3. N-PE	0.62***	0.77***	1***						
4. N-MBI-GS	0.88***	0.94***	0.9***	1***					
5. NF-Self-efficacy	−0.19***	−0.18***	−0.10**	−0.16***	1***				
6. NF-Hope	−0.13***	−0.10**	−0.02	−0.08*	0.79***	1***			
7. NF-Resilience	−0.12***	−0.09**	−0.02	−0.07	0.67***	0.84***	1***		
8. NF-Optimism	−0.13***	−0.10**	−0.03	−0.08*	0.67***	0.75***	0.79***	1***	
9. NF-PCQ	−0.14***	−0.11**	−0.02	−0.09*	0.85***	0.92***	0.92***	0.89***	1***

*** *p* < .01; ** *p* < .05; * p < .10

*N*- = Nurse, *NF*- = Nurse Family, *EE* = Emotional Exhaustion, *CY* = Cynicism, *PE* = Professional Efficacy

### Multiple linear regression analysis

The multiple linear regression model was designed based on theoretical frameworks and prior research, which suggest that PsyCap plays a critical role in influencing job burnout among nurses. Specifically, this study hypothesized that the PsyCap of nurses themselves, along with the PsyCap of nurse leaders and family members, would significantly affect burnout. While individual factors like self-efficacy and hope have been linked to lower burnout, external factors, such as leadership support and family encouragement, are expected to provide additional psychological resources, buffering burnout. Table 7 presents the multiple linear regression analysis predicting nurses’ MBI-GS scores. To ensure the validity of the regression model and the independence of the included variables, a multicollinearity check was used to conduct Variance Inflation Factors (VIF). All VIF values were found to be 1, indicating no multicollinearity issues. This confirms that the variables included in the model contribute independently, without redundancy, to the explanation of nurse burnout.

**Table 7:** Multiple linear regression analysis predicting N-MBI-GS total score

Variables	B	Std. Error	β	t	*p*	VIF	R^2^	Adjusted R^2^	F
Constant	76.99	13.00	–	5.92	0.000	–	0.21	0.20	F = 42.71***
N-PCQ	−0.44	0.04	−0.44	−10.86	0.000	1			
NL-PCQ	0.16	0.09	0.07	1.64	0.102	1			
NF-PCQ	−0.13	0.05	−0.11	−2.64	0.009	1			

*** p < .01; ** p < .05; * p < .10

Dependent Variable: N-MBI-GS

*N-* = Nurse, *NL-* = Nurse Leader, *NF-* = Nurse Family, *MBI-GS* = Maslach Burnout Inventory—General Survey, *PCQ* = Psychological Capital Questionnaire

The PsyCap scores of nurses were found to be a significant predictor of lower job burnout (β = −0.44, p < 0.01). The confidence interval for this coefficient was calculated to range from −0.52 to −0.36, indicating that we are 95% confident that the true population effect of PsyCap on burnout falls within this range. This reinforces the conclusion that higher PsyCap levels are associated with lower burnout. Nurse leaders’ PsyCap and family members’ PsyCap were included as control variables to examine their indirect effects on nurse burnout. While Nurse Leader PsyCap (β = 0.07, p = 0.102) had a weak direct effect on burnout, it was retained in the model due to its theoretical relevance in shaping supportive leadership behaviors. Nurse leaders play a critical role in influencing work environments and guiding nurses’ psychological resources through leadership behaviors. The PsyCap of nurses’ family members showed a significant negative effect on nurse job burnout (β = −0.13, p = 0.009). The 95% confidence interval for this coefficient ranged from −0.228 to −0.032, suggesting that we are 95% confident that the true effect of family PsyCap on burnout lies within this range, supporting the conclusion that higher family support is associated with lower levels of burnout among nurses.

### Mediation analysis

The mediation analysis results are detailed in Tables 8 and 9. Nurse PsyCap partially mediated the relationship between nurse leader PsyCap and nurse job burnout. Although the direct effect of nurse leader PsyCap on job burnout was weak (effect size = 0.17, p = 0.10), the indirect effect through nurse hope was significant (effect size = −0.03, p < 0.05). This finding underscores the importance of hope in mitigating burnout.

**Table 8:** Mediation analysis of nurse psychological capital in the relationship between nursing leader psychological capital and nurse job burnout

Effect	Path	Estimate	SE	*p*	95% CI	
					LL CI	UL CI
Direct Effect	NL-PCQ → N-MBI-GS	0.17	0.10	0.082*	−0.02	0.35
	NL-PCQ → N-Hope	−0.03	0.01	0.045**	−0.05	0.00
	N-Self-efficacy → N-Hope	0.77	0.02	0.000***	0.73	0.81
	N-Self-efficacy → N-Resilience	0.13	0.04	0.004***	0.04	0.21
	N-Hope → N-Resilience	0.70	0.05	0.000***	0.61	0.80
	N-Self-efficacy → N-Optimism	0.14	0.04	0.001***	0.06	0.22
	N-Hope → N-Optimism	0.18	0.05	0.001***	0.08	0.29
	N-Resilience → N-Optimism	0.65	0.04	0.000***	0.56	0.73
	N-Self-efficacy → N-MBI-GS	−0.64	0.28	0.025**	−1.19	−0.08
	N-Optimism → N-MBI-GS	−0.70	0.31	0.024**	−1.32	−0.09
Total Effect	NL-PCQ → N-MBI-GS	0.15	0.11	0.162	−0.06	0.36

*** p < .01; ** p < .05; * p < .10

*N-* = Nurse, *NL-* = Nurse Leader, *SE* = Standard Error, *CI* = Confidence Interval, *LL* = Lower Limit, *UL* = Upper Limit, *PCQ* = Psychological Capital Questionnaire, *MBI-GS* = Maslach Burnout Inventory—General Survey

**Table 9:** Mediation analysis of nurse psychological capital in the relationship between nurse family psychological capital and nurse job burnout

Effect	Path	Effect Size	SE	P	95% CI	
					LL CI	UL CI
Direct effect	NF-PCQ → N-MBI-GS	−0.13	0.05	0.010***	−0.22	−0.03
	N-Self-efficacy → N-Hope	0.76	0.02	0.000***	0.72	0.8
	N-Self-efficacy → N-Resilience	0.12	0.04	0.004***	0.04	0.21
	N-Hope → N-Resilience	0.7	0.05	0.000***	0.61	0.8
	N-Self-efficacy → N-Optimism	0.13	0.04	0.001***	0.05	0.21
	N-Hope → N-Optimism	0.19	0.05	0.001***	0.08	0.29
	N-Resilience → N-Optimism	0.65	0.04	0.000***	0.56	0.73
	N-Self-efficacy → N-MBI-GS	−0.55	0.28	0.049**	−1.1	0
	N-Hope → N-MBI-GS	−0.65	0.37	0.084*	−1.39	0.09
	N-Optimism → N-MBI-GS	−0.67	0.31	0.032**	−1.28	−0.06
Total effect	NF-PCQ → N-MBI-GS	−0.14	0.05	0.012**	−0.24	−0.03

*** p < .01; ** p < .05; * p < .10

*N-* = Nurse, *NF-* = Nurse Family, *SE* = Standard Error, *CI* = Confidence Interval, *LL* = Lower Limit, *UL* = Upper Limit, *PCQ* = Psychological Capital Questionnaire, *MBI-GS* = Maslach Burnout Inventory—General Survey

Similarly, the PsyCap of nurses’ family members had a significant direct negative effect on nurse job burnout (effect size = −0.13, p < 0.05). Indirect effects through nurse PsyCap components (e.g., self-efficacy, hope) further amplified this relationship. Among these, self-efficacy and optimism showed significant negative effects on burnout (p < 0.05), while resilience and hope had weaker or non-significant effects (p > 0.10).

## Discussion

In this study, 499 nurses, 108 nurse leaders, and 432 family members of nurses from three provincial and municipal hospitals and three township health centers in Zhejiang Province, China were analyzed on the correlation between PsyCap and nurse burnout. Through detailed descriptive statistics, correlation analyses, multiple linear regression analyses, and mediation analyses, the effects of PsyCap on nurse job burnout at three levels: individual nurses, nurse leaders, and family members of nurses were explored.

The results showed that most nurses reported some degree of job burnout, this distribution underscores the significant challenges facing the nursing profession, where a significant portion of the workforce is currently experiencing job burnout, which can adversely affect the well-being of nurses and the quality of patient care [25]. A higher prevalence of job burnout is associated with increased medical errors, decreased job satisfaction, and a higher propensity to leave the profession [26].

Significant demographic and professional variations in job burnout and PsyCap levels were observed. Nurses in township health centers reported higher burnout levels and lower PsyCap compared to those in provincial and municipal hospitals, likely reflecting differences in work environments and resource availability [27]. Female nurses exhibited higher burnout levels, aligning with prior research that links gender-specific stressors to higher emotional exhaustion in female nurses [28]. Additionally, younger and less experienced nurses were more vulnerable to burnout and had lower PsyCap, indicating that tenure and experience enhance resilience and coping abilities [29].

Consistent with existing literature, our correlation and regression analyses demonstrated a significant negative relationship between PsyCap and job burnout, confirming its protective role [30]. Hope and optimism emerged as the most influential PsyCap components, strongly associated with reduced burnout, in line with studies by Xie et al. [31]. The inclusion of nurse leaders’ PsyCap as a control variable highlighted its indirect role in shaping nurses’ psychological resources and mitigating burnout, consistent with findings by Muniba and Tehreem [32] and Sihvola et al. [33], which emphasize the importance of transformational leadership in fostering resilience. Furthermore, the PsyCap of nurses’ family members was found to influence nurse burnout, underscoring the interconnected nature of professional and personal dimensions [34]. Familial support emerged as a critical factor in alleviating workplace-induced stress [35].

The findings reinforce the critical role of nurses’ PsyCap in mitigating job burnout. While nurse leaders and family members may influence the work environment and personal well-being indirectly, the results highlight the need for targeted interventions that enhance nurses’ psychological resources directly.

Therefore, by focusing on nurses’ PsyCap, this study emphasizes its direct relationship with job burnout. Although nurse leaders and family members provide contextual support, their influence was considered secondary in this analysis.

These findings highlight the central role of nurses’ PsyCap in mitigating burnout. While leadership and familial influences provide contextual support, targeted interventions to enhance PsyCap at the individual level remain paramount. Developing strategies to strengthen PsyCap components, particularly hope and optimism, could significantly reduce burnout and improve overall nurse well-being.

### Limitations and suggestions

While this study provides valuable insights into the relationship between PsyCap and nurse job burnout, several limitations need to be acknowledged. First, the cross-sectional design limits the ability to establish causality. The directionality and temporal dynamics between PsyCap and burnout cannot be determined. Future research should consider longitudinal or experimental designs to better understand the causal mechanisms and long-term effects of PsyCap on burnout.

Second, the reliance on self-reported measures introduces the risk of biases such as social desirability and recall bias. To mitigate this, future studies could incorporate third-party assessments or objective measures to enhance the accuracy of self-reports and reduce bias.

Third, excluding participants with severe mental health conditions, while minimizing confounding effects, may limit the generalizability of the findings to nurses with pre-existing mental health challenges. Future research should explore how different levels of mental health conditions interact with PsyCap and influence burnout, to gain a deeper understanding of these relationships across diverse nurse populations.

Fourth, the geographic specificity of this study to Zhejiang Province may limit the generalizability of the findings to other regions with different cultural and organizational contexts. Expanding the research to include a broader range of geographical locations and healthcare systems would provide more generalizable insights into the role of PsyCap in nurse burnout.

Lastly, family members were recruited through nurses, which may have introduced self-selection bias, particularly overrepresenting those in supportive family relationships. Future studies should consider randomizing recruitment or employing independent selection methods. Additionally, factors such as organizational culture, peer support, and work-life balance, which were not measured in this study, could significantly contribute to burnout. A more comprehensive approach in future studies is needed to address these unmeasured variables and their impact on nurse well-being.

## Conclusion

This study examined the relationship between nurses’, nurse leaders’, and family members’ PsyCap and nurse job burnout at multiple levels. The findings indicated a significant negative association between nurses’ PsyCap and job burnout, suggesting that PsyCap may play a role in mitigating burnout. Additionally, the PsyCap of nurse leaders and family members appeared to have an indirect influence on nurse burnout, emphasizing the potential value of supportive work and familial environments.

These results offer insights for nursing management practices, particularly in considering PsyCap as a potential factor in burnout prevention. However, further research is needed to explore the mechanisms underlying PsyCap’s effects and to assess the impact of interventions targeting PsyCap development on nurse well-being.

## Data Availability

No datasets were generated or analysed during the current study.

## References

[1] Wei H. The development of an evidence-informed Convergent Care Theory: Working together to achieve optimal health outcomes. Int J Nurs Sci. 2022;9:11–25. 10.1016/J.IJNSS.2021.12.009. 35079601 10.1016/j.ijnss.2021.12.009 PMC8766786

[2] Nabizadeh-Gharghozar Z, Adib-Hajbaghery M, Bolandianbafghi S. Nurses’ job burnout: a hybrid concept analysis. J Caring Sci. 2020. 10.34172/JCS.2020.023. 32963984 10.34172/jcs.2020.023 PMC7492971

[3] Bailey AK, Sawyer AT, Robinson PS. A psychoeducational group intervention for nurses: rationale, theoretical framework, and development. J Am Psychiatr Nurses Assoc. 2023. 10.1177/10783903211001116. 34154451 10.1177/10783903211001116

[4] Babapour AR, Gahassab-Mozaffari N, Fathnezhad-Kazemi A. Nurses’ job stress and its impact on quality of life and caring behaviors: a cross-sectional study. BMC Nurs. 2022;21:1–10. 10.1186/S12912-022-00852-Y/TABLES/5. 35361204 10.1186/s12912-022-00852-y PMC8968092

[5] Sun F, Wang A, Xue J, Su J, Hu C, Lu Q. The mediating effect of psychological capital on the relationship between psychological stress and distress among chinese nursing students: a cross-sectional study. BMC Nurs. 2022. 10.1186/S12912-022-00915-0. 35614502 10.1186/s12912-022-00915-0 PMC9130981

[6] Yan D, Wen F, Li X, Zhang Y. The relationship between psychological capital and innovation behaviour in Chinese nurses. J Nurs Manag. 2020. 10.1111/JONM.12926. 31811781 10.1111/jonm.12926

[7] Shoman Y, Marca SC, Bianchi R, Godderis L, Van Der Molen HF, Guseva CI. Psychometric properties of burnout measures: a systematic review. Epidemiol Psychiatr Sci. 2021. 10.1017/S2045796020001134. 33436137 10.1017/S2045796020001134 PMC8057391

[8] Yıldırım N, Coşkun H, Polat Ş. The relationship between psychological capital and the occupational psychologic risks of nurses: the mediation role of compassion satisfaction. J Nurs Scholarsh. 2021;53:115–25. 10.1111/JNU.12607. 33146952 10.1111/jnu.12607

[9] Tang Y, Wang Y, Zhou H, Wang J, Zhang R, Lu Q. The relationship between psychiatric nurses’ perceived organizational support and job burnout: mediating role of psychological capital. Front Psychol. 2023;14:1099687. 10.3389/FPSYG.2023.1099687/BIBTEX. 36895741 10.3389/fpsyg.2023.1099687 PMC9989200

[10] Wei H, Roberts P, Strickler J, Corbett RW. Nurse leaders’ strategies to foster nurse resilience. J Nurs Manag. 2019;27:681–7. 10.1111/JONM.12736. 30449038 10.1111/jonm.12736

[11] Suban Hoda F, Sani Mahoklory S, Ketzia Pello Y, Falde Riti I, Stik MK, et al. Association between family support and nurse motivation in managing patients with COVID-19. IJNP Indones J Nurs Pract. 2021;5:123–31. 10.1819/IJNP.V5I2.13352.

[12] Naing L, Bin NR, Abdul RH. Sample size calculation for prevalence studies using Scalex and ScalaR calculators. BMC Med Res Methodol. 2022. 10.1186/S12874-022-01694-7. 35907796 10.1186/s12874-022-01694-7 PMC9338613

[13] Gang L, Ni S, Xiaoling C. A Meta-analysis of the detection rate of nurses’ job burnout in China. Occup Health. 2022;38:1854–9.

[14] Ahmed F, Xiong Z, Faraz NA, Arslan A. The interplay between servant leadership, psychological safety, trust in a leader and burnout: assessing causal relationships through a three-wave longitudinal study. Int J Occup Saf Ergon. 2022. 10.1080/10803548.2022.2086755. 35678558 10.1080/10803548.2022.2086755

[15] Chaoping Li. Translation developing the human competitive edge : Psychological capital. Beijing: China Light Industry Press; 2008.

[16] Luthans F, Avey JB, Clapp-Smith R, Li W. More evidence on the value of Chinese workers’ psychological capital: a potentially unlimited competitive resource? Int J Hum Resour Manag. 2008;19:818–27. 10.1080/09585190801991194.

[17] Ren Z, Zhang X, Li X, He M, Shi H, Zhao H, et al. Relationships of organisational justice, psychological capital and professional identity with job burnout among Chinese nurses: a cross-sectional study. J Clin Nurs. 2021;30:2912–23. 10.1111/jocn.15797. 33829587 10.1111/jocn.15797

[18] Zhang M, Chen H, Wang N, Li Y, Li X, Liu Y. The mediating role of job satisfaction between psychological capital and work engagement among Chinese nurses during COVID-19 outbreak: A comparative study between nurse specialists and general nurses. Front Psychiatry. 2023. 10.3389/FPSYT.2022.990216/PDF. 36713893 10.3389/fpsyt.2022.990216 PMC9878697

[19] Xiao S, Shi L, Lin H, Zhao S, Ou W, Zhang J, et al. The impact of psychological capital on turnover intention among Chinese nurses: a moderated mediation model. J Nurs Manag. 2022;30:3031–40. 10.1111/jonm.13702. 35661464 10.1111/jonm.13702

[20] Wilmar Schaufeli, Michael Leiter, Christina Maslach, Susan E. Jackson. Maslach Burnout Inventory -- General Survey (GS) . Maslach Burnout Inventory Manual 1996.

[21] Chaoping Li, Kan S. The influence of distributive justice and procedural justice on job burnout. Acta Psychologica Sinic. 2003;3:677–84.

[22] Kalimo R, Pahkin K, Mutanen P, Toppinen-Tanner S. Staying well or burning out at work: Work characteristics and personal resources as long-term predictors. Work Stress. 2003;17:109–22. 10.1080/0267837031000149919.

[23] Luo A, Kong W, He H, Li Y, Xie W. Status and influencing factors of social media addiction in Chinese medical care professionals: a cross-sectional survey. Front Psychol. 2022. 10.3389/FPSYG.2022.888714/PDF. 35572263 10.3389/fpsyg.2022.888714 PMC9097152

[24] Wu L, Ren L, Wang Y, Zhang K, Fang P, Liu X, et al. The item network and domain network of burnout in Chinese nurses. BMC Nurs. 2021;20:147. 10.1186/s12912-021-00670-8. 34404401 10.1186/s12912-021-00670-8 PMC8369754

[25] Xia M, Wang J, Bi D, He C, Mao H, Liu X, et al. Predictors of job burnout among Chinese nurses: a systematic review based on big data analysis. Biotechnol Genet Eng Rev. 2023. 10.1080/02648725.2023.2168910. 36683283 10.1080/02648725.2023.2168910

[26] Im C, Song S, Kim K. The associations of psychological burnout and time factors on medication errors in rotating shift nurses in Korea: a cross sectional descriptive study. Nurs Open. 2023;10:5550–9. 10.1002/NOP2.1794. 37115503 10.1002/nop2.1794 PMC10333822

[27] Rees CS, Eley R, Osseiran-Moisson R, Francis K, Cusack L, Heritage B, et al. Individual and environmental determinants of burnout among nurses. J Health Serv Res Policy. 2019;24:191–200. 10.1177/1355819619840373. 31291766 10.1177/1355819619840373

[28] D’ettorre G, Pellicani V, Vullo A. Gender assessment of job stress in healthcare workers Implications for practice. Med Lav. 2019. 10.2374/MDL.V110I1.7421. 30794245 10.23749/mdl.v110i1.7421 PMC7810005

[29] Najafi B, Nasiri A. Explaining novice nurses’ experience of weak professional confidence: a qualitative study. SAGE Open Nurs. 2023. 10.1177/23779608231153457. 36969365 10.1177/23779608231153457 PMC10031601

[30] Luthans F, Youssef C, Avolio B. Psychological capital and beyond. USA: Oxford University Press; 2015.

[31] Yang Z, Zhang M, Guo Y, Wang R, Xie F. Burnout among nurses: a bibliometric analysis of the global publications. Psychol Res Behav Manag. 2024;17:1727–39. 10.2147/PRBM.S458199. 38681974 10.2147/PRBM.S458199 PMC11055547

[32] Muniba ML, Tehreem TA. The role of transformational leadership and PsyCapin shaping organizational citizenship behavior: exploring job satisfaction as a mediator. Int J Manag Res Emerg Sci. 2024;14:182–200. 10.5653/IJMRES.V14I3.664.

[33] Sihvola S, Kvist T, Nurmeksela A. Nurse leaders’ resilience and their role in supporting nurses’ resilience during the COVID-19 pandemic: a scoping review. J Nurs Manag. 2022;30:1869–80. 10.1111/JONM.13640. 35434873 10.1111/jonm.13640 PMC9115204

[34] Yuan L, Nguyen TTN, Vu MC. Transformational leadership and its impact on performance: The role of psychological capital and collectivism. ACM Int Conf Proc Series. 2018. 10.1145/3180374.3181325.

[35] Thomas PA, Liu H, Umberson D. Family relationships and well-being. Innov Aging. 2017. 10.1093/GERONI/IGX025. 29795792 10.1093/geroni/igx025 PMC5954612

